# Estimation of diffuse Cherenkov optical emission from external beam radiation build-up in tissue

**DOI:** 10.1117/1.JBO.26.9.098003

**Published:** 2021-09-20

**Authors:** Savannah M. Decker, Daniel A. Alexander, Rachael L. Hachadorian, Rongxiao Zhang, David J. Gladstone, Petr Bruza, Brian W. Pogue

**Affiliations:** a Dartmouth College, Thayer School of Engineering, Hanover, New Hampshire, United States; b Dartmouth College, Geisel School of Medicine, Department of Medicine, Hanover, New Hampshire, United States; c Norris Cotton Cancer Center, Dartmouth–Hitchcock Medical Center, Lebanon, New Hampshire, United States; d DoseOptics LLC, Lebanon, New Hampshire, United States

**Keywords:** Cherenkov, surface dose, in-vivo dosimetry

## Abstract

**Significance:** Optical imaging of Cherenkov emission during radiation therapy could be used to verify dose delivery in real-time if a more comprehensive quantitative understanding of the factors affecting emission intensity could be developed.

**Aim:** This study aims to explore the change in diffuse Cherenkov emission intensity with x-ray beam energy from irradiated tissue, both theoretically and experimentally.

**Approach:** Derivation of the emitted Cherenkov signal was achieved using diffusion theory, and experimental studies with 6 to 18 MV energy x-rays were performed in tissue phantoms to confirm the model predictions as related to the radiation build-up factor with depth into tissue.

**Results:** Irradiation at lower x-ray energies results in a greater surface dose and higher build-up slope, which results in a ∼46% greater diffusely emitted Cherenkov signal per unit dose at 6 MV relative to 18 MV x-rays. However, this phenomenon competes with a decrease in signal from less Cherenkov photons being generated at lower energies, a ∼44% reduction at 6 versus 18 MV. The result is an emitted Cherenkov signal that is nearly constant with beam energy.

**Conclusions:** This study explains why the observed Cherenkov emission from tissue is not a strong function of beam energy, despite the known strong correlation between Cherenkov intensity and particle energy in the absence of build-up and scattering effects.

## 1 Introduction

**Fig. 1:**
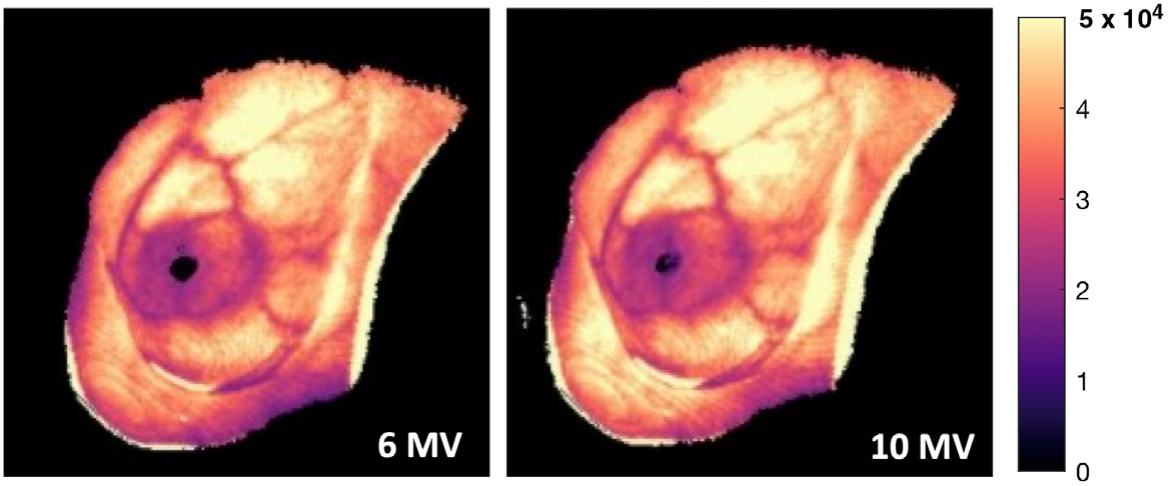
Comparison of cumulative Cherenkov images taken during whole-breast radiation therapy at 6 versus 10 MV x-rays for a single patient.^6^ The observed Cherenkov intensity remains approximately constant, even at higher x-ray energies.

Cherenkov light emission occurs in patient tissues during radiation therapy, and the development of imaging systems to selectively capture this signal has paved a way to visualize the treatment delivery in real time.^1^^–^^4^ The ability to visualize the beam shape is well documented, although the interpretation of the emitted intensity is much more complex because of the range of factors that influence the observed signal intensity.^5^^,^^6^ While several studies have examined the influence of tissue optical properties, few have systematically examined the nature of the radiation beam itself in affecting the observed emitted Cherenkov signal intensity. In particular, there has always been a mystery about why the intensity of Cherenkov light emitted from patients undergoing radiation therapy does not appear to be a strong function of beam energy, despite the fact that it is well known that the basic production of Cherenkov light is a strong function of particle energy^7^ (Fig. 1). Cherenkov light is continuously emitted from secondary electrons as they scatter inside tissue and are emitted over a range of angles. In this paper, both analytic theory and experimental validation studies are used to interpret the diffuse Cherenkov radiant emission from tissue, relevant to changes in radiation therapy beam characteristics and interactions with tissue optical properties.

Cherenkov emission increases with the energy of a radiation beam, with the threshold for emission near the x-ray energy of 220 keV.^8^ However, every x-ray beam has a broad energy spectrum, which is affected by the beam settings and beam size/shape.^5^ Earlier studies have shown that the energy spectrum of the incident ionizing radiation greatly affects the magnitude of Cherenkov emission^5^ and alters the Cherenkov/dose value because higher x-ray energies produce more Cherenkov light per unit dose.^7^ One of the more perplexing issues has been that the entrance of radiation into tissue induces a build-up effect that is energy spectrum specific, and it is this dose deposition with increasing depth that influences the Cherenkov emission. In this study, the phenomenon of dose per unit depth was explicitly examined for the first time as a factor in the observed Cherenkov emission.

The other major factor influencing the observed Cherenkov emission in radiation therapy is the optical absorption and scattering inherent within tissue. In particular, blood attenuation is quite visible, as major blood vessels are highly attenuating and alterations in subcutaneous layer composition can change the radiant emission drastically.^6^ This optical attenuation effect has been examined in a number of theoretical,^5^^,^^7^^–^^10^ as well as experimental,^6^ studies. The competing effects of radiation build-up combined with optical attenuation must be grappled with and are folded into the theoretical analysis here, based upon diffusion theory modeling.^11^^,^^12^ Briefly, the therapeutic x-ray beam can be treated as the source for Cherenkov light generation within tissue, which is then transported by the tissue scattering, and the radiant emission is determined as a function of beam and tissue properties. These effects are examined here theoretically and then validated experimentally.

## 2 Theory

Cherenkov light is continuously emitted during high energy radiation interactions as the cascade of radiation dose happens through secondary particle emission. The directions of the electrons emitting Cherenkov are randomized somewhat at each interaction step, and the Cherenkov light itself is highly scattered in tissue with a scattering length near 50 to 200 microns, depending upon wavelength. Thus, Cherenkov light emitted from the surface of human tissue is predominantly highly diffused, randomized photons. The modeling work below starts with the assumption that all light emitted from the surface comes from highly scattered light described by diffusion theory.

### 2.1 Optical Diffusion Theory

**Fig. 2:**
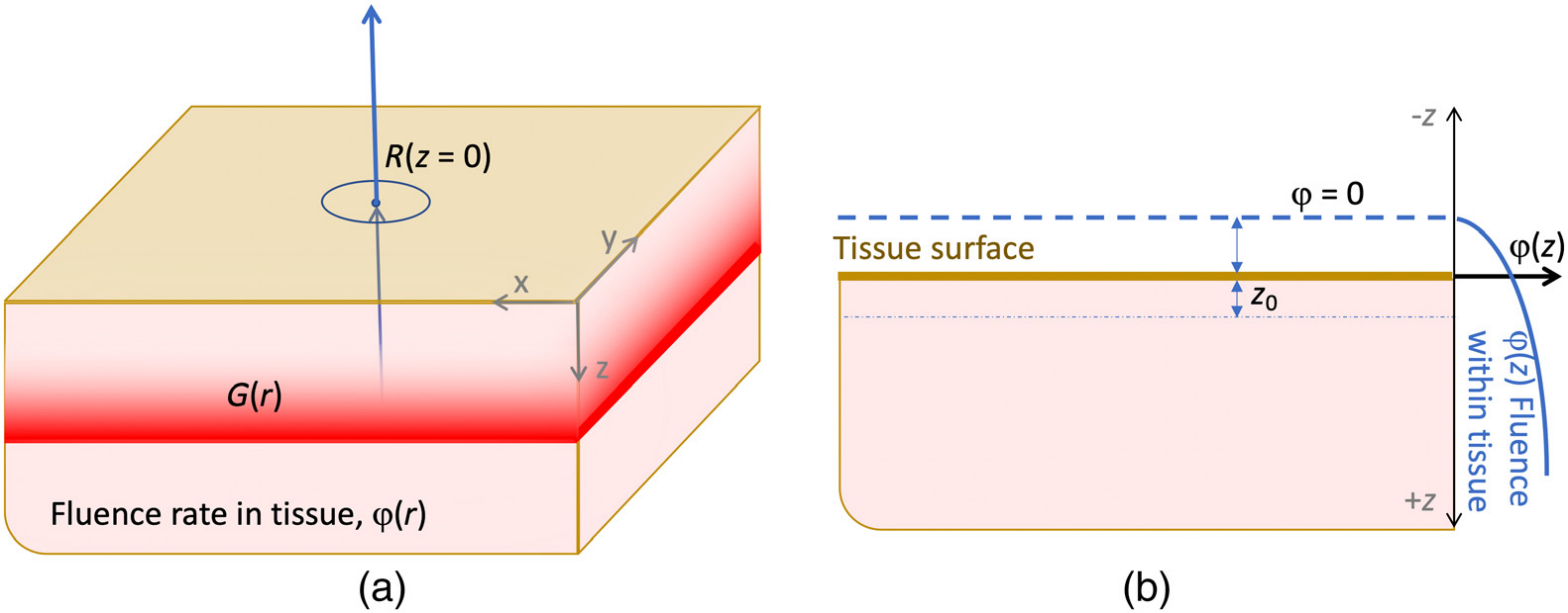
The geometry of a slab of tissue is shown (a) with orthogonal axes (x,y,z) and radiant emission R(z=0) out of the top surface, in the −z direction. The geometry to estimate internal fluence with the extrapolated boundary method is shown in (b) with the assumption that at some distance above the surface, the fluence drops to zero. $z_{0}$ represents the depth of the apparent source, S(r).

The total radiant emission of Cherenkov light from tissue, R, can be approximated as the diffuse emitted light that has been diffused by scattering interactions and escapes the surface. As there is exponential attenuation of non-scattered light by the total attenuation coefficient of tissue, $\mu_{t}\approx 100\;{\mathrm{cm}}^{-1}$, after a few hundred microns, the light is fully diffused, meaning that the original directionality of the photons is lost due to multiple scattering. Here, we assume that all light that escapes the surface is fully diffuse. For highly scattered light, attenuation in tissue over distances greater than a few millimeters can be reasonably accurately described by the diffusion equation ^11^:

$$-D\nabla^{2}\varphi (r)+\mu_{a}\varphi (r)=S(r),$$ (1)

where the geometry is shown in Fig. 2(a), and here, D is the diffusion coefficient and $\mu_{a}$ is the tissue absorption coefficient. The homogeneous tissue reduced scattering coefficient is $\mu_{s}^{\prime }$, which defines $D=1/3{\mu^{\prime }}_{s}$. The source of light inside the tissue (the primary x-ray beam) is described by S(r), and this equation is used to solve for the isotropic fluence rate of the light inside the medium, φ(r), at any location, r=(x,y,z). In terms of light emission from the tissue surface, the radiant emission, R(x,y), exiting the surface can be defined as the gradient of the fluence rate exiting the surface (at z=0), which is simply the spatial derivative of the internal fluence rate as it flows out of the surface, as shown in Fig. 2(b), given by Fick’s Law:

$$R=-D{\frac{\partial \varphi (r)}{\partial z}|}_{z=0}.$$ (2)

Therefore, to solve for the diffuse fluence rate, the solution to Eq. (1) for φ(r) can be used.

To predict a light distribution, the diffusion theory functional form solution to Eq. (1), G(r), becomes the kernel for transport away from the source, so it needs to be convolved with the source distribution to accurately reflect the light field from an extended source, as

**Fig. 3:**
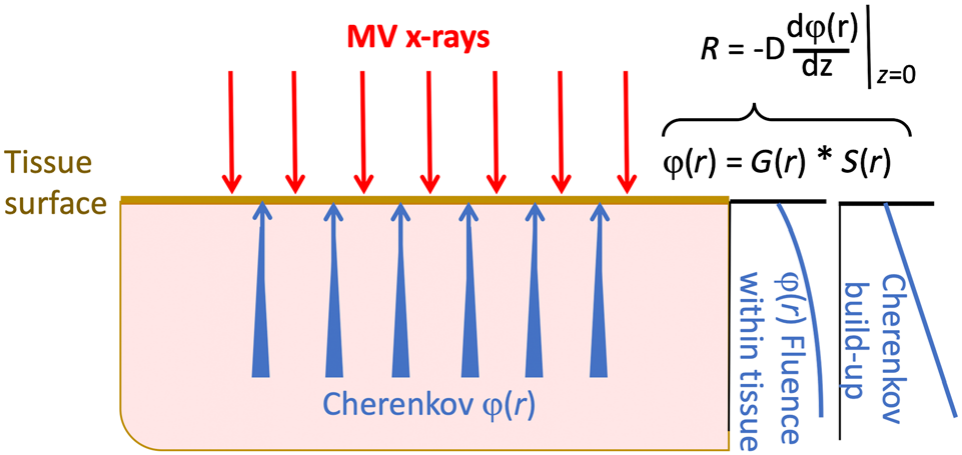
Conceptually, the emitted Cherenkov light from tissue is a convolution of the Cherenkov generation with the probability of escape from the upper surface. This latter probability is dictated by the attenuation by tissue optical interaction coefficients, predicted from the diffusion equation over moderate distances.

φ(r)=G(r)*S(r), (3)

or

$$\varphi (r)=\int_{0}^{\infty }G(r-r^{\prime })S(r^{\prime })dr^{\prime },$$ (4)

in terms of a general planar source solution to Eq. (1). In the case of Cherenkov emission, the light generation follows the dose build-up since Cherenkov emission is assumed to be directly proportional to dose.^1^ One way to think of this is that the escaping light is predicted by the diffuse fluence, which will be decreasing with proximity to the surface, and that this is convolved with the generation of Cherenkov light, from the dose delivery source of Cherenkov, S(r). This is visually shown in Fig. 3.

### 2.2 X-Ray Radiation Dose & Build-Up as the Source of Cherenkov

**Fig. 4:**
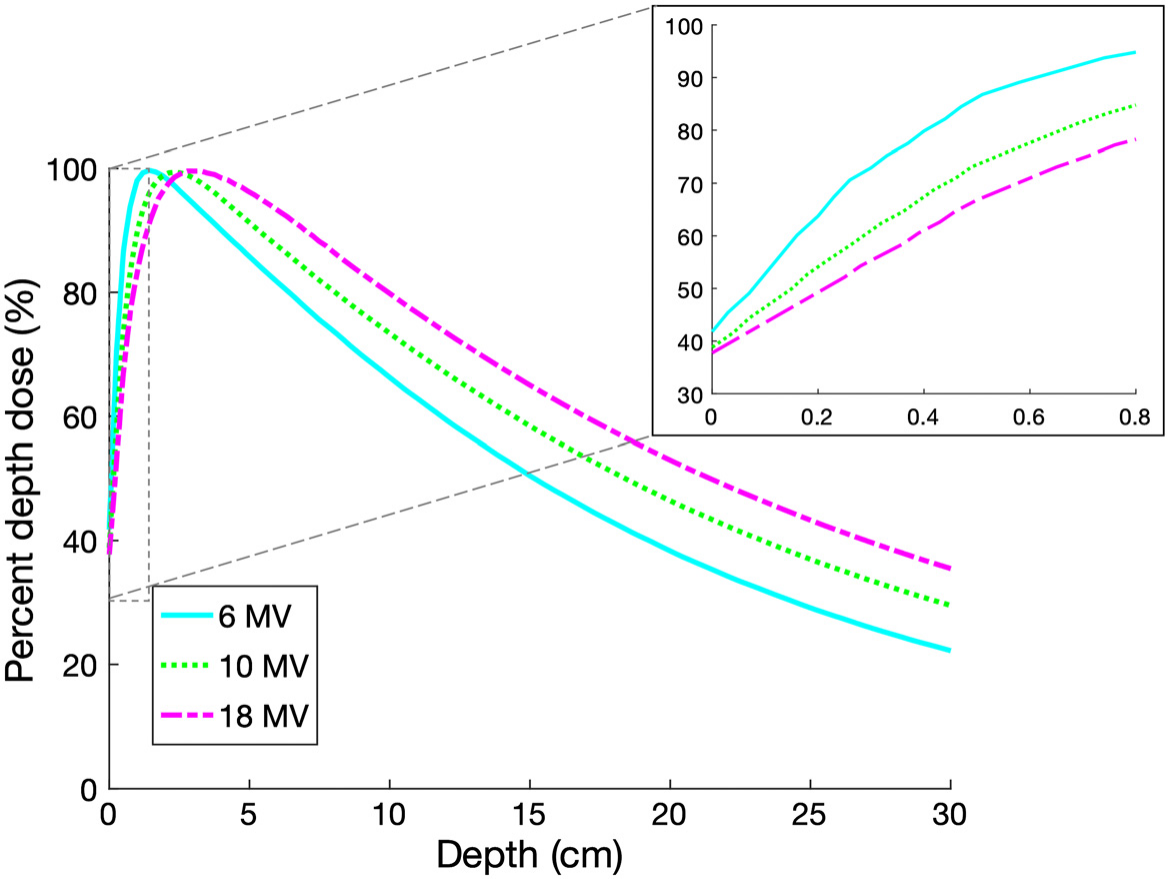
The TPS calculated PDD curves are shown for photon beams at SSD=100  cm. The first 0.8 cm of dose deposition contains the only relevant contribution to Cherenkov light that can be emitted from tissue, given that deeper Cherenkov light is largely attenuated as it exits the surface. For each energy, a linear approximation is quite accurate in this shallow depth range.

As in the approximations stated above, the source of optical photons, S(r), could be assumed to be directly proportional to the dose deposited by a specific megavoltage beam with a given energy spectrum, assuming that beam hardening effects are negligible. Typical depth dose measurements show that these shallow depths are dominated by the build-up region, where the dose is below the maximum, and builds up to $d_{\max}$, the point of maximum dose deposition, which is larger than the limit of Cherenkov detection depth. In Fig. 4, the Eclipse treatment planning system (TPS) percent depth dose (PDD) curves, calculated using the Analytical Anisotropic Algorithm (Varian Medical Systems, Palo Alto, California) are displayed over the full range of 6, 10, and 18 MV x-ray beams over a depth of 30 cm, and in the first 0.8 cm where the build-up region is nearly linear.^13^ These plots are typical and are affected by factors such as the source-to-surface distance (SSD), beam diameter, variations in collimator scatter, and irradiated volume backscatter. However, in general, for a water-equivalent material, the dose increases approximately linearly within a depth smaller than 0.5 $d_{\max}$, owing to the charge particle disequilibrium.

In this study, where Cherenkov emission is known to only escape from tissue within the top 0.4 to 0.8 cm based upon Monte Carlo simulations,^14^ we use the dose deposition inside the tissue to approximate the Cherenkov source equation increasing with depth,

$$S(z)=\{\begin{array}{ll} k_{1}+k_{2}z & 0<z<0.8\;\mathrm{cm}\\ 0 & \text{otherwise} \end{array}.$$ (5)

These two k values vary considerably with beam energy, beam size, and material composition, and so are specific to the curves in Fig. 4. These are well known parameters for most irradiation geometries and can be obtained from annual PDD verification data. Therefore, for beams wider than a few centimeters in diameter, this would be an approximation. Notably, $k_{1}$ represents the dose at the surface and $k_{2}$ is proportional to the gradient of the build-up with depth. From Fig. 4, for clinically relevant beams, $k_{1}$ ranges between 0.35 and 0.45 and $k_{2}$ ranges between 0.5 to $0.7\;{\mathrm{cm}}^{-1}$.

While this definition of the source function is physically representative of the generation of Cherenkov photons within the tissue, it poses a mathematical issue for the definition of the radiant emission, R, given by Fick’s Law in Eq. (2). R is proportional to the spatial derivative of φ(r) at z=0. However, φ(r) is discontinuous at this point, and therefore nondifferentiable, and so a common approach to solving this issue is to use the extrapolated boundary method to formulating the diffusion solution. While this approach has a long history of developing the formalism, it can be simplified by taking the derivative inside the boundary where the fluence exists.^15^^–^^17^

### 2.3 Diffuse Cherenkov Emission in One Dimension

For the specific case of a broad planar irradiation of tissue, this theory approximates a 1D situation. The 1D diffusion equation may be used, and assuming the tissue is homogenous in x and y, the equations above simplify significantly. Equation (1) therefore becomes:

$$-D\frac{\partial^{2}\varphi (z)}{\partial z^{2}}+\mu_{a}\varphi (z)=S(z),$$ (6)

or

$$\frac{\partial^{2}\varphi (z)}{\partial z^{2}}-\mu_{f}^{2}\varphi (z)=-\frac{S(z)}{D},$$ (7)

where $\mu_{\mathrm{eff}}={\left(\mu_{a}/D\right)}^{\frac{1}{2}}$ is the effective attenuation coefficient of the tissue. This equation has the following general solution:^18^

$$G(z)=\frac{1}{2D\mu_{\mathrm{eff}}}e^{-\mu_{\mathrm{eff}}z}.$$ (8)

The convolution in Eq. (3) with the estimates from Eqs. (5) and (8) then yields:

$$\varphi (z)=(\frac{1}{2D\mu_{\mathrm{eff}}}e^{-\mu_{\mathrm{eff}}(z)})*(k_{1}+k_{2}z).$$ (9)

**Fig. 5:**
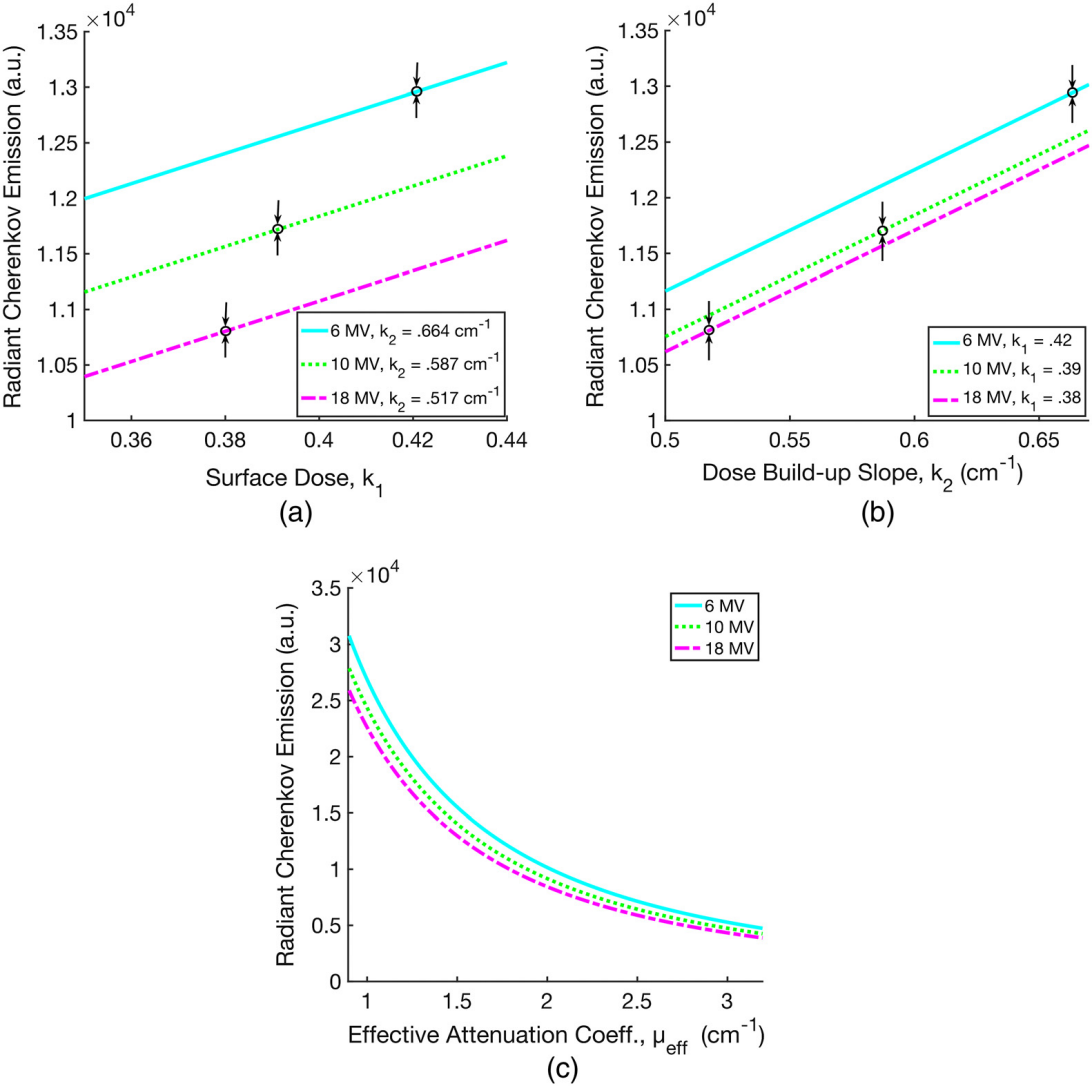
The calculated radiant Cherenkov emission from Eqs. (2) and (9) is plotted as a function of surface dose (a), dose build-up slope (b), and the effective attenuation coefficient in tissue (c). In (a), $k_{1}$ is varied with constant $k_{2}$ values for 6, 10, and 18 MV x-ray beams as indicated. The black arrows denote the specific $k_{1}$ and $k_{2}$ values for the corresponding energies. Figure (b) is analogous to (a), with $k_{2}$ varied over three constant $k_{1}$ values for 6, 10, and 18 MV.

Combining Eq. (9) with Fick’s Law in Eq. (2), the radiant Cherenkov emission, R, from the tissue surface at z=0 is evaluated as a function of $k_{1}$ and $k_{2}$, and this is plotted in Figs. 5(a) and 5(b). For tissue, where $\mu_{a}=0.1\;{\mathrm{cm}}^{-1}$ and ${\mu^{\prime }}_{s}=10.0\;{\mathrm{cm}}^{-1}$, we approximate the optical coefficients as $D=1/3{\mu^{\prime }}_{s}\approx 0.033\;\mathrm{cm}$, and $\mu_{\mathrm{eff}}\approx 1.7\;{\mathrm{cm}}^{-1}$ in the near infrared. Figure 5(c) shows the relationship between R and $\mu_{\mathrm{eff}}$ for each x-ray beam energy over a practical human tissue range, 0.9 to $3.2\;{\mathrm{cm}}^{-1}$. Figure 5(c) provides insight into the origins of the signal emitted at the surface, accounting both for beam energy and tissue optical properties. It is worth noting that the derivation of R does not account for more Cherenkov photons generated at higher energies.^7^

## 3 Materials and Methods

### 3.1 Imaging Irradiated Tissue Phantoms

Diffuse liquid tissue phantoms were made with a combination of water, bovine blood, and Intralipid^®^ to simulate the optical properties of human tissue.^19^ The concentration of Intralipid^®^ varied between 0.5% and 2%, in steps of 0. 25%, whereas the concentration of blood varied between 0.5% and 3.5%, in steps of 0.5%. These values were chosen to reflect the range of optical scattering and absorption properties for various human tissue types. The tissue phantoms were contained in a black, open-top, plastic cylinder of depth 1.9 cm, inner diameter 8.8 cm, and outer diameter 9.1 cm when irradiated.

**Fig. 6:**
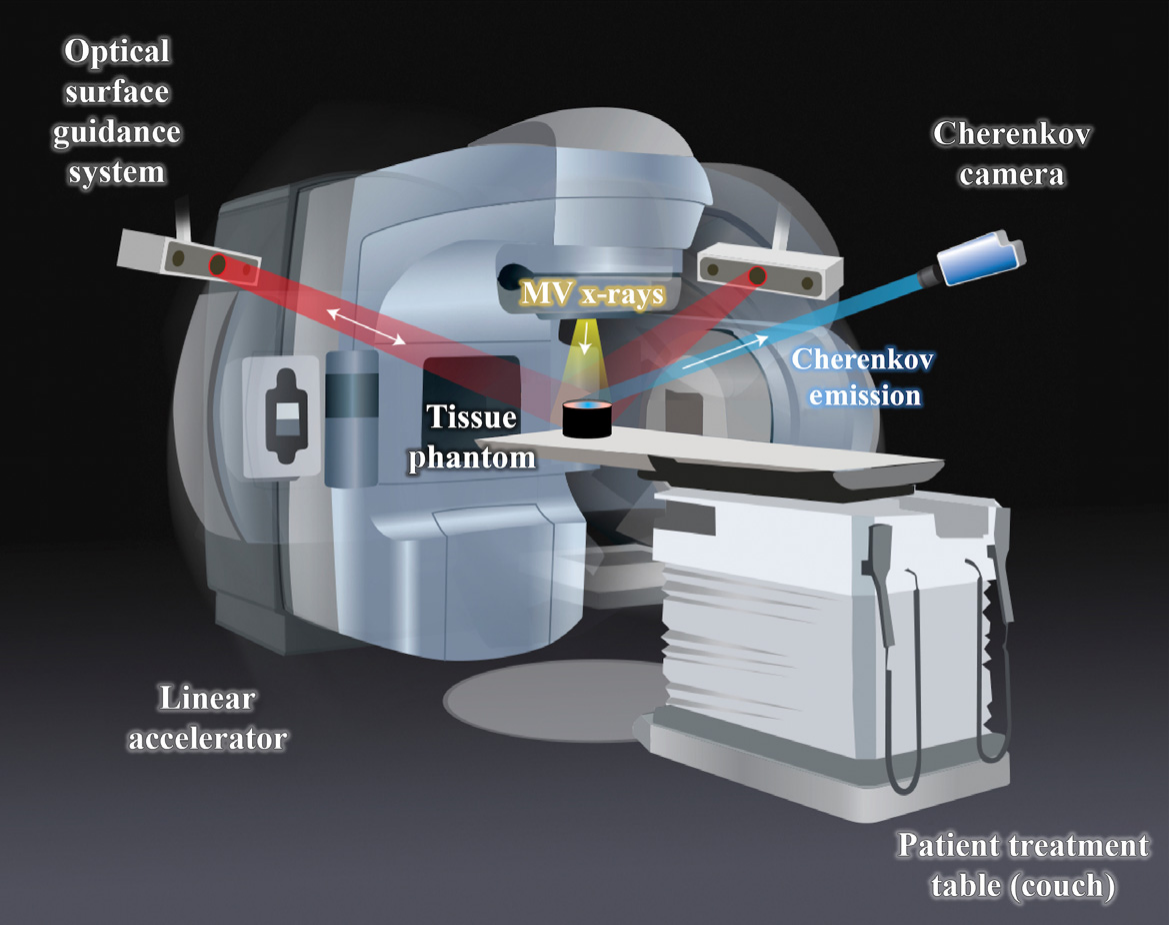
Illustration of experimental setup. The linear accelerator irradiates a tissue phantom placed on the couch with MV x-rays, and the gantry can rotate around the couch. The Cherenkov camera fixed to the ceiling captures the Cherenkov emission from the phantom. The clinical setup also includes an optical surface guidance system that is not utilized during these measurements.

A TrueBeam linear accelerator (Varian Medical Systems, Palo Alto, California) was used to irradiate the tissue phantoms with 200 MU using 6, 10, and 18 MV x-rays, shown in Fig. 6. The SSD was kept at a constant 100 cm, and the field-size was ${10\times 10\;\mathrm{cm}}^{2}$. To measure the radiant Cherenkov emission from the entrance surface where the x-ray beam penetrates the phantom, the gantry was positioned top-down at 0 deg. To measure the Cherenkov emission from the exit surface, opposite to where the beam penetrates the phantom, the gantry was positioned at 180 deg. Data collection was performed using an intensified CMOS camera (C-Dose Research, DoseOptics LLC, Lebanon NH) fixed with a 50-mm f/2.8 lens (Nikkor 50 mm, Nikon, Tokyo Japan). The image intensifier in the camera used a red-sensitive photocathode, as described by Alexander et al.^20^ The camera remained fixed to the ceiling for each imaging acquisition, and the room lights were turned off the minimize external noise.

**Fig. 7:**
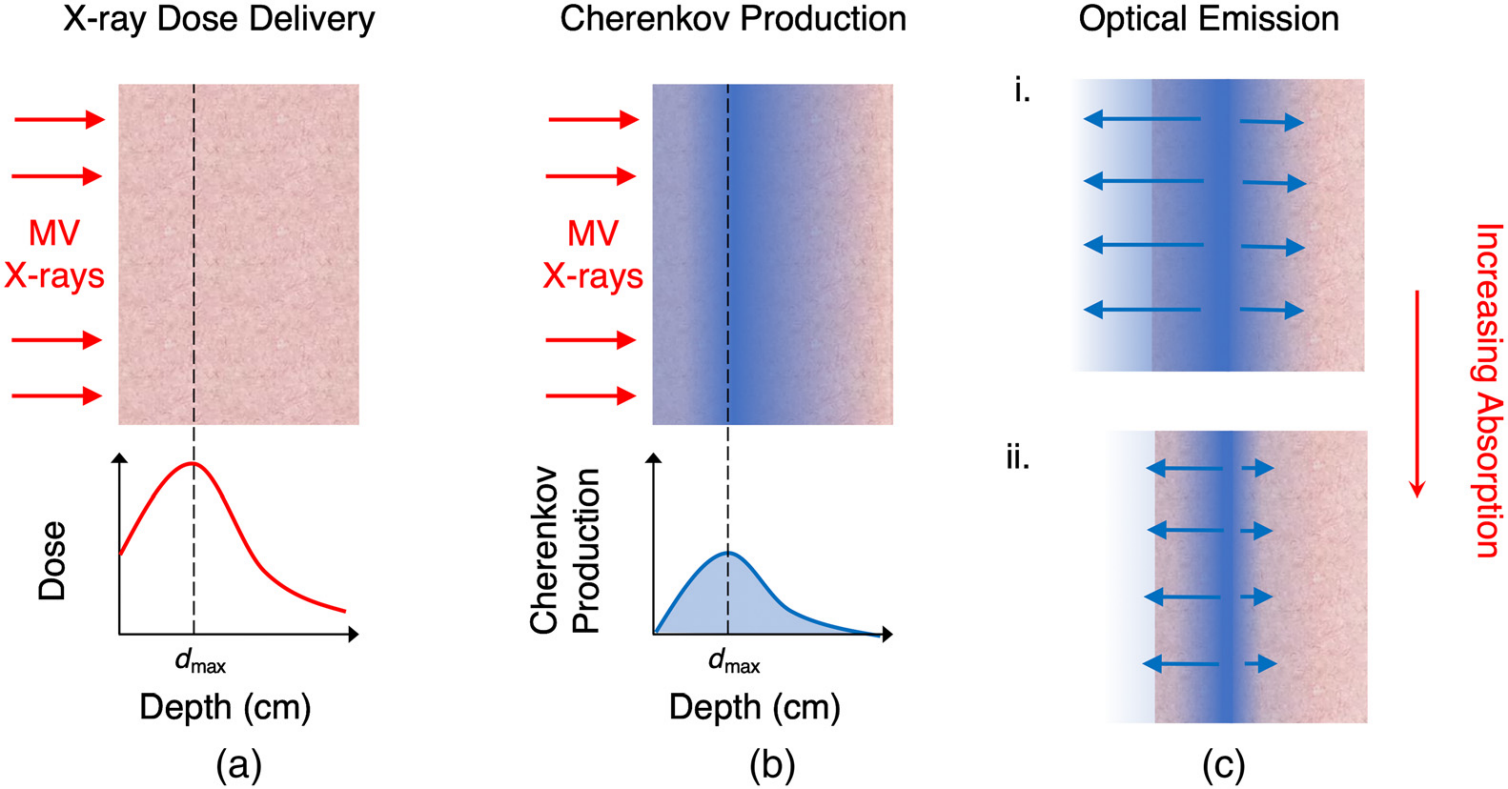
Illustration of what is being measured by the camera. MV x-rays are delivered to a tissue phantom (a) and deposit dose as a function of depth, represented qualitatively by the graph. The depth at maximum dose, $d_{\max}$, is also the point of maximum Cherenkov light production (b). As tissue optical properties vary, so does the amount of light that exits the surface. As the tissue absorption increases, less Cherenkov light makes it out of the phantom (c).

To image the emission from the exit surface, $d_{\max}$ was approximated using the PDD profiles in Fig. 4. For each x-ray energy, the phantom depth was effectively adjusted to the height of the corresponding $d_{\max}$ by adding water-equivalent slabs underneath the phantom container at the beam entrance surface. Entrance and exit surface images were then taken at these depths for each Intralipid^®^/blood concentration. Figure 7 illustrates the x-ray dose delivery, Cherenkov light generation, and optical emission from the tissue.

### 3.2 Data Processing

Image data acquired by the CMOS camera was processed and displayed with C-Dose software (C-Dose Research, DoseOptics LLC, Lebanon NH). All image analysis was conducted via MATLAB Version R2020b (MathWorks, Natick, Massachusetts). For each image acquisition, all Cherenkov frames were summed into a cumulative image^6^ and flat-field corrected.^20^ The radiant Cherenkov emission was then determined by taking the mean pixel intensity of a rectangular region-of-interest (ROI) at the center of each irradiated phantom. The slopes of the PDD curves were approximated by applying a linear least squares regression between 0 and 0.8 cm using MATLAB’s curve fitting tool.

## 4 Results

### 4.1 Comparison of Entrance and Exit Surface Cherenkov Emission

**Fig. 8:**
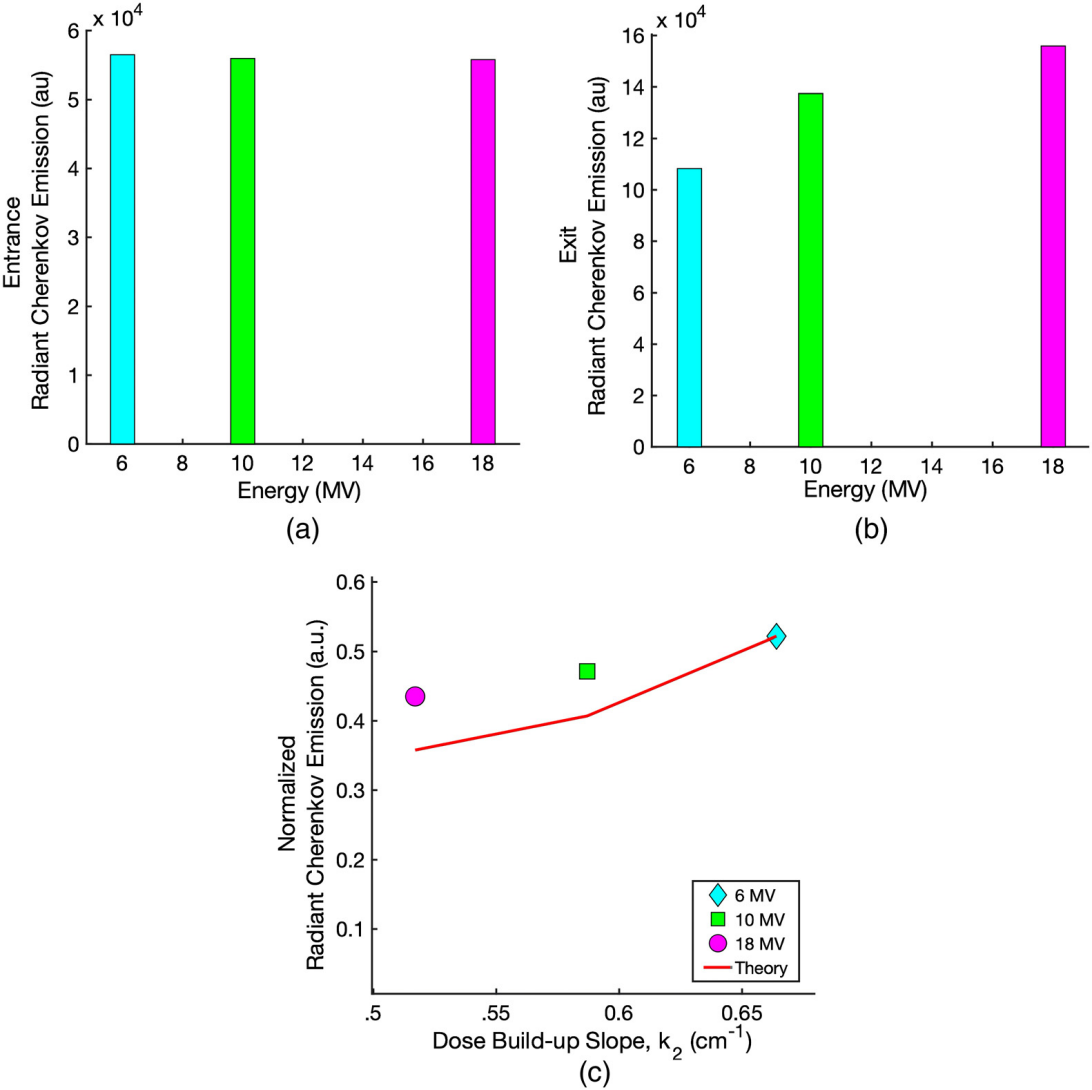
The Cherenkov emission from the entrance surface (a) and the exit surface (b) of the 1% blood/1% Intralipid^®^ diffuse liquid tissue phantom as a function of x-ray energy. The measurements were taken over the full range of energies on the Varian TrueBeam linear accelerator. Figure (c) shows the entrance Cherenkov emission normalized by the relative exit Cherenkov emission plotted as a function of the slopes of the Varian Eclipse TPS PDD data. Figure (c) also shows the calculated values of R from Sec. 2.3, which predicts the normalized Cherenkov light emission from the entrance surface.

The 1% blood/1% Intralipid^®^ diffuse liquid tissue phantom was designed to match the typical absorption and scattering properties of soft human tissue. This was irradiated to compare the intensity of the radiant Cherenkov emission out of the beam entrance surface to that emitted out of the beam exit surface at $d_{\max}$. Figure 8(a) shows the Cherenkov emission intensity measured out of the entrance surface for 6, 10, and 18 MV x-rays. Over this range of clinically significant energies, the radiant Cherenkov emission from the entrance surface is approximately constant, deviating no more than 1.3% between 6 and 18 MV. The radiant Cherenkov emission from the exit surface is plotted in Fig. 8(b). Overall, the Cherenkov emission from the exit surface at $d_{\max}$ is ∼2× to 3× greater than that which escapes the entrance surface due to less tissue attenuation.

At the exit surface, the radiant Cherenkov emission increases ∼44% with increasing x-ray energy from 6 to 18 MV, as the amount of Cherenkov photons generated within the tissue phantoms increases.^7^ For this reason, the exit surface Cherenkov emission is taken to be approximately proportional to the amount of Cherenkov photons generated. Thus, to isolate the effect of the slope of the dose build-up on the Cherenkov emission from the entrance surface, the entrance surface values are normalized by the corresponding exit surface values and plotted as a function of $k_{2}$, shown in Fig. 8(c). At 6 MV, the normalized radiant Cherenkov emission is ∼46% greater than at 18 MV. These values are then compared to the theoretical derivation for the radiant Cherenkov emission from the surface as a function of $k_{2}$. The calculated values were normalized such that R at 6 MV ($k_{2}=0.664\;{\mathrm{cm}}^{-1}$) is equal to the normalized measured Cherenkov emission at 6 MV. For the normalized measured Cherenkov emission at 10 and 18 MV, this leads to a 15% and 20% deviation, respectively, from the theoretical predictions of R.

### 4.2 Varying Absorption and Scattering Properties

**Fig. 9:**
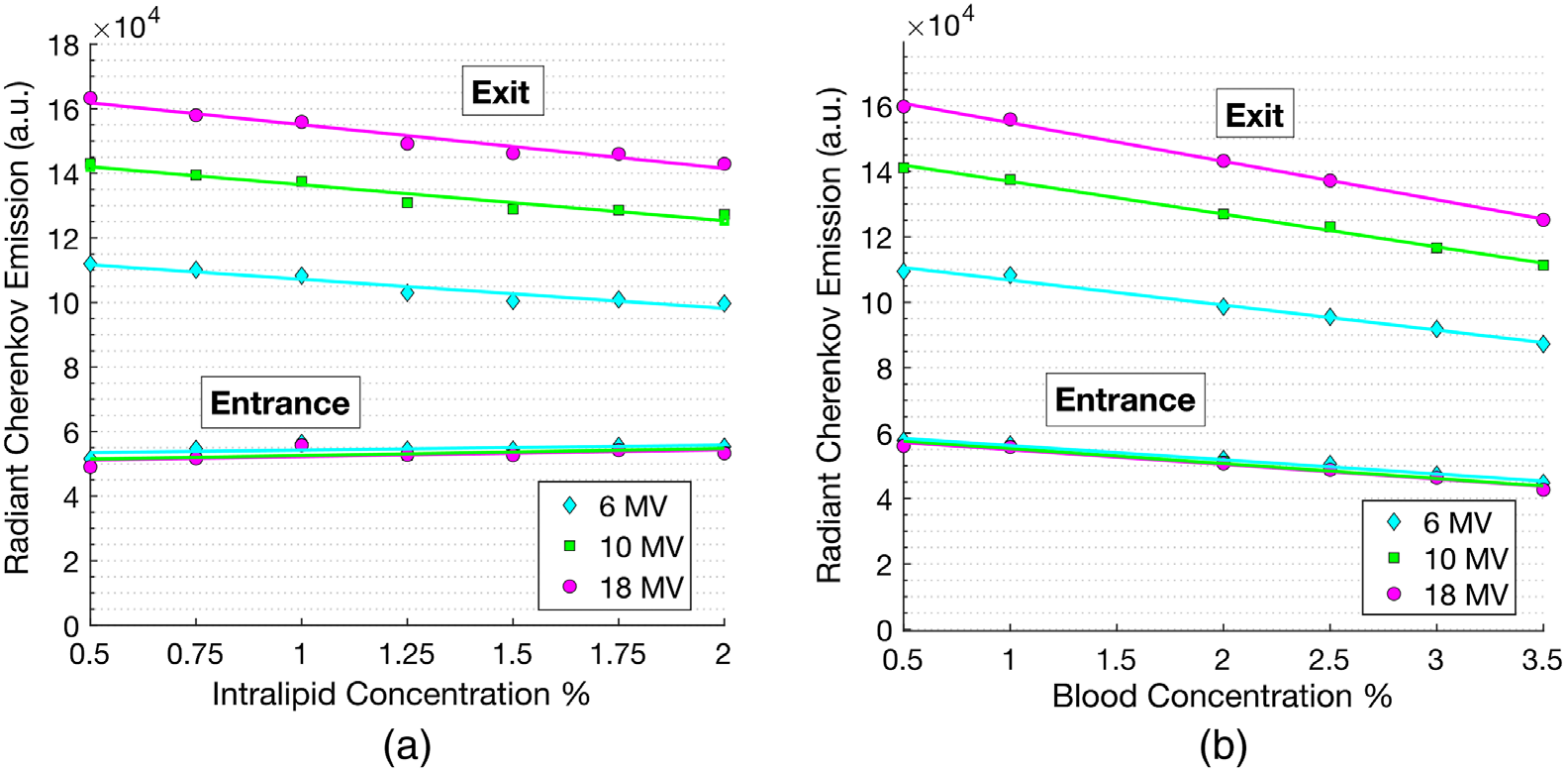
Radiant Cherenkov emission as a function of Intralipid^®^ concentration (scattering) is shown in (a) and blood concentration (absorption) is shown in (b). Each figure displays the Cherenkov emission at both the exit and entrance surfaces. All measurements were taken over the range of linear accelerator x-ray energies: 6, 10, and 18 MV.

Figures 9(a) and 9(b) show the radiant Cherenkov emission measured at the exit and entrance surfaces as a function of Intralipid^®^ and blood concentrations for 6, 10, and 18 MV x-ray beams. Increasing the concentration of Intralipid^®^ effectively increased the tissue reduced scattering coefficient, ${\mu^{\prime }}_{s}$. Measured at the exit surface, the Cherenkov emission decreased with increased scattering by ∼10% over the full range of measurements. At the entrance surface, the radiant Cherenkov emission increased with increasing Intralipid^®^ concentration by ∼10%. Increasing the bovine blood concentration effectively demonstrated how the Cherenkov emission changes with increasing the absorption coefficient, $\mu_{a}$. Varying the blood concentration from 0.5% to 3.5% had ∼20% reduction effect for both the exit and entrance measurements. With both increasing blood and Intralipid concentrations, a main discrepancy between the opposing surface measurements is that at the entrance surface, varying x-ray energy did not have an effect on the Cherenkov emission as the optical properties of the phantom changed. At the exit surface, however, a higher x-ray energy routinely produced greater Cherenkov emission.

## 5 Discussion

Following a theoretical derivation of the radiant Cherenkov emission from the surface of x-ray irradiated tissue, several factors affecting this signal were examined experimentally. Irradiating diffuse liquid phantoms that simulate the range of scattering and absorption properties of human tissue with 6, 10, and 18 MV x-rays provided insight into the magnitude at which these factors affect the Cherenkov light that is ultimately emitted from the surface.

At lower energies, not only is the surface dose greater, but the slope of the dose build-up is also steeper as the beams do not penetrate as deeply into tissue, leading to a smaller build-up region to $d_{\max}$ and consequently a greater dose build-up slope, shown in Fig. 4. However, these factors are offset by the generation of more Cherenkov photons at higher beam energies. Due to these effects both influencing the Cherenkov light that is ultimately emitted from the surface of opposing, yet nearly equivalent, magnitudes, the result is a constant signal at the entrance surface displayed over the range of clinical beam energies.

When measuring at the exit surface at $d_{\max}$, adjusting the depth of the liquid tissue phantoms introduced a build-down region. As the height of a phantom was adjusted such that the exit surface was at the point of $d_{\max}$, there was no tissue past that point to contribute back-scattered electrons to the maximum dose deposited, as is typically the case in clinical scenarios. Consequently, this alters the PDD profile and therefore the actual $d_{\max}$. However, Monte Carlo simulations showed that the relative change in dose deposition near the exit surface varies only slightly between different energies, ∼0.3%, when the build-down region is introduced. Thus, the measurements of the Cherenkov emission from the exit surface are assumed to accurately reflect the Cherenkov intensity at $d_{\max}$.

As the scattering was increased with various phantoms, the measured Cherenkov emission from the entrance surface increased, contrary to what the theory expressly states. One possible explanation for this effect originates in the approximation that all light escaping the surface is highly diffuse. Within the first few hundred microns, the x-ray photons have directionality down into the tissue. Consequently, the secondary electrons generated in this region are more likely to travel down into the tissue as well and generate Cherenkov photons in a downward cone around their paths. Thus, as the Intralipid^®^ concentration is increased, these near-surface Cherenkov photons are more likely to scatter in the opposite direction, namely toward the entrance surface, and possibly contribute to increased Cherenkov emission at that surface.

Overall, the results of measuring the radiant Cherenkov emission at the entrance and exit surfaces with varying tissue optical properties were consistent with the results from plotting the 1% blood/1% Intralipid^®^ phantom Cherenkov emission, where the measured entrance surface emission did not vary with x-ray energy, yet the exit intensities increased with increasing x-ray energy. Though the theoretical derivation of R versus $\mu_{\mathrm{eff}}$ predicted a greater emitted signal at lower energies because it did not account for varying Cherenkov photon generation, the nearly constant relative difference in Cherenkov emission between energies over varying tissue optical properties is consistent with our results.

The observation that tissue optical properties can be used to provide a linear correction to Cherenkov light emission has been studied recently with a system that can specifically image regions of tissue to estimate the absorption and scattering coefficients.^6^ While it has yet to be applied, this approach provided a way to correct for attenuation due to superficial blood vessels and regions of tissue that vary in these properties. Earlier Monte Carlo studies indicated that linear corrections were possible as well,^5^ and because the observation there indicates that it is the product of the absorption and scattering coefficients, or the square of the effective attenuation coefficient, it is comparatively easy to measure this parameter from tissue using a number of lower technology reflectance approaches. Overall, the attenuation due to tissue optical properties is more significant than variations in Cherenkov production from beam build-up or energy changes.

## 6 Conclusions

By using diffusion theory solutions integrated with a source function that is a linear approximation to the radiation build-up over the first 0.8 cm of tissue, an expression for the radiant Cherenkov emission was derived. The Cherenkov light emitted from tissue is inversely proportional to the product of the absorption and reduced scattering coefficients of the tissue, and linearly proportional to both the surface dose and the slope of the PDD build-up. The magnitudes of these effects were demonstrated experimentally by irradiating diffuse liquid tissue phantoms of varying optical properties with several clinical x-ray energies. While this derivation is limited to broad beam areas and homogeneous tissue regions, this observation has been documented in previous experimental studies. The interpretation of this work suggests that linear corrections to the Cherenkov emission based upon tissue interaction coefficients may be possible, allowing a quantitative calibration of the Cherenkov light emitted from tissue as being proportional to the dose delivered in superficial tissue layers. Further analysis may be done with more extensive analysis of beam size effects and with corrections to tissue optical properties to understand the effects of areas of non-radiation equilibrium and tissue property heterogeneity.
